# Editorial: Evolution of mitral valve disease treatment: from surgery to transcatheter therapy

**DOI:** 10.3389/fcvm.2024.1369596

**Published:** 2024-01-25

**Authors:** Antonio Lio, Francesco Loreni, Antonio Miceli, Dominik Wiedemann

**Affiliations:** ^1^ Department of Cardiac Surgery and Heart Transplantation, San Camillo Forlanini Hospital, Rome, Italy; ^2^ Department of Cardiac Surgery, Istituto Clinico Sant’Ambrogio, Milan, Italy; ^3^ Department of Cardiac Surgery, University Hospital of St. Pölten, St. Polten, Austria; ^4^ Department of Cardiac Surgery, Medical University of Vienna, St. Polten, Austria

**Keywords:** mitral valve, mitral valve repair (MV repair), mitral valve replacement (MVR), transcatheter mitral valve (MV) repair, transcatheter mitral valve implantation (TMVI)

**Editorial on the Research Topic** Evolution of mitral valve disease treatment: from surgery to transcatheter therapy

## Introduction

The mitral valve (MV) apparatus is a complex, and dynamic structure, whose alteration can significantly impact the functioning of the cardiovascular system (1). Mitral regurgitation (MR) affects approximately 2.5 million individuals in the United States and Europe, making it the most common valvular pathology in clinical practice (2–5). In recent times, surgical approaches to the MV have undergone extraordinary evolution. Starting from traditional median sternotomy, we have developed a spectrum of minimally invasive options, ranging from the ministernotomy over minithoracotomy accesses up to totally endoscopic options, such as robotic surgery. More recently, percutaneous treatment has emerged as an important alternative to surgery, especially in high-risk patients and in functional MR (FMR), where results of surgery are not so robust as in degenerative MR. This topic will explore some of these new techniques, sparking scientific discussions that are essential for advancing and improving these innovative methods.

## Papers presentation

Surgery has been the treatment of choice for MR for many years. The primary goal of the recent innovations is to reduce the invasiveness of the standard sternotomy treatment. Many papers have shown several advantages of minimally invasive treatment of MV disease. In this field, robotic treatment of MR is the latest innovation. In their paper, Massey et al. discuss about robotic MV repair: this approach exhibits a quicker recovery period with shorter hospital stays, lower morbidity, and comparable mid-term durability and mortality when compared to sternotomy. The global count of robotic cardiac surgical procedures will continue to increase as proficiency with robotic techniques grows and the review examines both the advantages and disadvantages of robotic MV surgery, considering crucial technical details related to both the operational setup and MV repair techniques.

Although surgical results for degenerative MR treatment are excellent, a great debate remains about FMR. Nowadays, surgery for isolated FMR is still indicated in case of aortic valve (AV) or coronary artery disease treatment. In this setting, Asher et al. analyzed the trends in concomitant mitral surgery with aortocoronary bypass or AV replacement. In their study, 1,515 patients were included. Simultaneous MV repair or replacement did not affect the risk of postoperative mortality for patients with moderate MR or more than moderate MR. In patients with more than moderate MR undergoing only aortocoronary bypass, there was a survival advantage from simultaneous MV repair or replacement in the first two postoperative years, but a higher incidence of subsequent MV interventions in the five subsequent years was observed.

As stated before, percutaneous treatment of MR has recently emerged as an important alternative especially in patients with high surgical risk and FMR. The first percutaneous technology that has been introduced was the transcatheter edge-to-edge repair (TEER): the numbers of TEER procedures have significantly increased over the last years and FMR emerged as a main indication for TEER. After the two major trials (MITRA-FR and COAPT) a huge discussion was ongoing because of conflicting results: Mitra-FR has showed no improvement in prognosis over medical therapy while in the COAPT trial the MitraClip procedure reduced the rates of hospitalization for heart failure and mortality. These differences may be explained by a disproportionately higher MR in the COAP trial; moreover, compared with patients in the COAPT trial, those enrolled in the MITRA-FR trial had substantially more left ventricular (LV) damage, with severe LV dilatation/dysfunction (6, 7). In their work, Felbel et al. compared short-term and one-year outcomes in patients with FMR undergoing TEER or surgical MV repair (SMVr). A meta-analysis was performed, including 21 studies on TEER and 37 on SMVr. In-hospital mortality was significantly lower with TEER, and 1-year mortality did not differ significantly. The recent paper from Stone et al. has confirmed the low rate of short-term mortality of the TEER procedure, but long-term mortality in FMR patients with impaired ventricular function is still considerable with a 5-year mortality close to 60% (8).

Moving from the TEER, many percutaneous options for MV replacement have been introduced; Alperi et al. conducted a systematic review of the published literature providing clinical data on transcatheter MV replacement (TMVR), evaluating short- and mid-term outcomes. A total of 347 patients were included. The 30-day mortality, stroke, and major bleeding rates were 8.4%, 2.6%, and 15.6%, respectively; there was a statistically significant reduction in ≥grade 3+ MR and in the number of patients exhibiting poor functional class after the intervention.

Given the continuous development of new transcatheter technologies and the data emerging from published data, it's possible to imagine that transcatheter will gradually expand to treat lower-risk patients, as previously seen for AV disease treatment. However, the highly variable on complex spectrum of MV pathologies are a far greater challenge to address in terms of device engineering than in AV disease. In this setting, a multidisciplinary approach is crucial to define the correct treatment option; the paper from Cocchieri et al. fits into this area, outlining the technical prerequisites and local constraints crucial for optimizing interventions on the MV. A significant body of literature delineates the range of indications and treatment expectations for Transcatheter MV Intervention (TMVI), incorporating procedures such as TEER and TMVR. The expected performance of TMVI should be assessed based on the established standards of risk-benefit considerations and actual outcomes derived from minimally invasive MV surgery. At the institutional level, cardiac teams can make informed decisions by drawing upon the knowledge acquired from surgical experiences in MV disease.

Clinical imaging has a crucial importance for transcatheter therapy: the development of imaging modalities during the last decades have been tremendous and will contribute vastly to the field of MV surgery and interventions. Nowadays the field in general is on the move away from a purely risk patient decision making pathway towards a more anatomy-based decision making. Wong et al. have examined how clinical imaging modalities can facilitate the creation of specific models of the MV with high precision, accurately replicating the MV's pathophysiology. Pre-procedural computational modeling will play a crucial role in guiding physicians toward optimal interventions; additionally, collaborative efforts to develop MV models will contribute to establishing atlases of pathologies and biomechanical profiles, outlining which patient groups would derive greater benefit from specific surgical options compared to TMVR (Table 1).

**Table 1:** Selected articles on the topic.

Authors	Title	Keywords	Summary
**Massey et al.**	Robotic mitral valve surgery	• Mitral valve • Robotic • Minimally invasive • Mitral valve repair • Outcomes	Both the advantages and disadvantages of robotic mitral valve surgery will be discussed, with a focus on important technical details concerning both the preparatory phase of the operation and mitral valve repair techniques
**Asher et al.**	Effect of concurrent mitral valve surgery for secondary mitral regurgitation upon mortality after aortic valve replacement or coronary artery bypass surgery	• Mitral valve surgery • Aortic valve surgery • Coronary artery bypass surgery • Mortality • Survival	The obtained results align with the 2021 European guidelines, which recommend intervention during coronary artery bypass graft for severe secondary mitral regurgitation. However, the data do not support the 2020 American guidelines suggesting mitral valve repair or replacement concurrent with aortic valve replacement, with or without coronary artery bypass graft
**Felbel et al.**	Comparison of transcatheter edge-to-edge and surgical repair in patients with functional mitral regurgitation using a meta-analytic approach	• Functional mitral regurgitation • Transcatheter edge-to-edge repair • Surgical mitral valve repair • Heart failure • Mitraclip	Hospital mortality was significantly lower in patients with functional mitral regurgitation (FMR) treated with TEER, while an increase in one-year mortality was observed in this high-risk population
**Alperi et al.**	Current status of transcatheter mitral valve replacement: systematic review and meta-analysis	• Mitral valve • Mitral regurgitation • Mitral insufficiency • Mitral incompetence • Transcatheter mitral valve replacement	Among 12 studies and 347 patients, a statistically significant reduction in ≥grade 3+ mitral regurgitation was observed. The overall rate of major bleeding emerged as the main drawback of this technique
**Cocchieri et al.**	Technical prerequisites and local boundary conditions for optimization of mitral valve interventions-emphasis on skills development and institutional risk performance	• Mitral • Minimally • Invasive • Percutaneous • Risk • Reduction • TMVR • MIMVS	Adapting to local conditions in terms of infrastructure, referrals, and reimbursement appears to be essential for the development of a comprehensive mitral valve disease management program. The effectiveness of Institutional Risk Management Performance (IRMP) and team skills should be integrated into an appropriate infrastructure that allows scalability and provides complete and secure solutions for mitral valve diseases
**Wong et al.**	Looking towards the future: patient specific computational modeling to optimize outcomes for transcatheter mitral valve repair	• Mitral valve • Transcatheter mitral valve repair • Computational modeling • Finite element • Fluid-structure Interaction patient specific model	The use of clinical imaging will allow the creation of patient-specific models with high precision and the ability to replicate mitral valve pathophysiology. It is anticipated that TMVR technology will gradually expand to treat lower-risk patient groups, and thus, pre-procedural computational modeling will play a crucial role in guiding operators toward optimal intervention

## Conclusion

This topic seeks to clarify the latest evidence for the treatment of MV pathology. We emphasize that both surgical repair/replacement and interventional techniques are highly valid procedures. However, it is crucial to choose the right technique based on the individual clinical and physical characteristics of the patient.
